# Immune Response of *Bos indicus* Cattle against the Anti-Tick Antigen Bm91 Derived from Local *Rhipicephalus (Boophilus) microplus* Ticks and Its Effect on Tick Reproduction under Natural Infestation

**DOI:** 10.1155/2012/907607

**Published:** 2012-11-19

**Authors:** Christian Lambertz, Natthaphon Chongkasikit, Sathaporn Jittapalapong, Matthias Gauly

**Affiliations:** ^1^Department of Animal Science, Georg-August University, Albrecht-Thaer-Weg 3, 37075 Göttingen, Germany; ^2^Department of Animal and Aquatic Science, Chiang Mai University, Chiang Mai 50200, Thailand; ^3^Department of Parasitology, Faculty of Veterinary Medicine, Kasetsart University, Bangkok 10903, Thailand

## Abstract

Antigens of anti-tick vaccines are more efficacious for homologous challenge with local tick strains. cDNA clones encoding for Bm91 from local *Rhipicephalus (Boophilus) microplus* strains were developed to immunize *Bos indicus* cattle under field conditions. Three groups of six animals each were injected with the antigen Bm91, saline, and adjuvant, respectively. Animals were immunized three times at 3-week intervals and a fourth time after six months. The anti-Bm91 antibody level, measured by ELISA, was monitored for 7 months and the reproductive performance of naturally infested *R. (B.) microplus* was determined. Bm91-immunized animals developed a strong immune response expressed by high anti-Bm91 levels remaining on high levels until the end of the study. Western blot analysis confirmed that Bm91 is immunogenic. Compared to control animals, the reproductive efficiency index and the egg viability were 6% and 8%, respectively, lower in the Bm91 group (*P* < 0.05). In conclusion, it was demonstrated that Bm91 induced a long-lasting immune response. However, the effect on the tick reproduction was not sufficient for an efficient tick control. Further studies under field conditions are warranted to enhance the effect on the tick reproduction by optimizing the immunization regimen, alone or in combination with other vaccine candidate antigens.

## 1. Introduction

*Rhipicephalus (Boophilus) microplus *is one of the most prevalent tick species in the world and causes tremendous economic losses for livestock producers in tropical and subtropical regions. Tick infestations have adverse physiological effects on the host and result in decreased live weight gains [1]. Beside these direct effects, ticks harm their hosts indirectly by transmitting tick-borne pathogens, such as *Babesia bovis, Babesia bigemina, Theileria annulata, *and* Anaplasma marginale *[2]. Global losses of tick infestations and tick-borne diseases have been estimated to more than US$ 18 billion [3]. By impacting cattle production to such an extent, tick control is a continuing global priority. It relies largely on the application of chemical acaricides. Depending on location and farming system, costs for the chemical control of ticks have been estimated between US$ 2.50 and US$ 25 per animal per year [4]. However, these drugs have limited efficacy in reducing tick infestations and are accompanied by serious drawbacks including the selection for acaricide-resistant ticks, environmental contamination, contamination of animal products with drug residues, and high costs for farmers [5]. Especially the selection of acaricide-resistant ticks reinforces the need for alternative approaches to control ticks more sustainable. From different regions of the world *R. (B.) microplus* populations resistant to the most commonly used acaricides, namely, the macrocyclic lactone ivermectin [6–8], the organophosphate diazinon [9], the amidine amitraz [10], and other organophosphates as well as synthetic pyrethroids [10, 11] have been reported.

Alternative tick control methods including pasture spelling, predators and parasites, and artificial selection for tick-resistant cattle can reduce tick burdens [12], but the enhancement of host resistance through immunization would constitute a major advance in order to reduce the reliance on acaricides. Based on the midgut antigen Bm86 two commercial vaccines against *R. (B.) microplus* were registered in Australia (TickGARD) and Cuba (Gavac) in the early 1990's [13, 14]. Several other tick-protective antigens were isolated. One of these is the salivary and midgut glycoprotein Bm91 [15], which has many biochemical and enzymatic properties in common with the mammalian angiotensin-converting enzyme [16]. Anti-tick vaccines are based on the concept of “concealed” antigens. During tick feeding the antigens are not exposed to the host immune system and therefore are not adapted for evasion of the host immune response. They can therefore invoke a protective immune response to the ticks by inducing the production of specific immunoglobulins [17]. The major effect of these vaccines is a successive reduction in tick numbers through reduced tick fertility. In immunization trials with artificial tick infestations substantial reductions of the number of engorging ticks, the tick weight, and the tick fertility were found and led to overall vaccine efficiencies of 45–90% [18–21]. However, strain-to-strain sequence variations in the antigen locus between *R. (B.) microplus *isolates from different geographical regions have been shown to influence the vaccine efficacy. For the sequence of Bm86 a 3.4% divergence at the amino acid level was reported by García-García et al. [22]. The authors suggested that variations in the amino acid sequence exceeding 2.8% would be sufficient to cause vaccine inefficiencies. In South American strains divergences of up to 6.1% and 4.6% in the sequence of the Bm86 and Bm95 protein, respectively, were found by Sossai et al. [23]. 

The specific objectives of this study were (1) to measure the humoral immune response induced by Bm91 in *Bos indicus *cattle under field conditions, (2) to confirm that antibodies invoked by Bm91 bind to *R. (B.) microplus* proteins, and (3) to assess the efficacy of Bm91 on the reproductive performance of naturally infested *R. (B.) microplus* ticks. 

## 2. Materials and Methods

### 2.1. Animals and Farming System

The study was conducted at the Department of Animal and Aquatic Science of the Chiang Mai University, Thailand (18° 47′ N, 98° 59′ E; 312 m above sea level). The climate is tropical with a rainy (June to October), winter (November to February), and summer season (March to May). Average daily temperature and rainfall are 25.9°C and 1,197 mm. The experiment lasted from April 2009 until October 2009. Eighteen purebred females of an indigenous *Bos indicus *cattle breed (White Lamphun) with an average age of 3 years and 103 ± 17 kg live weight were used. The animals were randomly allocated to the three groups Bm91, control, and adjuvant with 6 animals each. Only nonpregnant heifers were used to reduce an error, which may be introduced as a result of pregnancy status. The animals were raised in an extensive free-grazing system and grazing was allowed at all times, concentrate was not supplemented, and water was available ad libitum. During the preceding six months the animals had not been treated with acaricides. Rectal temperature was measured weekly and body weight fortnightly.

### 2.2. Immunizations

Animals in the Bm91-immunized group were inoculated with a vaccine formulation based on the recombinant Bm91 antigen, which was derived from a *R. (B.) microplus *strain indigenous to Thailand. The antigen was produced by recombinant DNA technology as previously described by Kaewhom et al. [24]. Briefly, mRNA was isolated from salivary glands of the ticks and cDNA encoding for the Bm91 sequence was synthesized by reverse transcriptase PCR (RT-PCR). The cDNA encoding for the Thai strain Bm91 protein was transferred to the *P. pastoris *plasmid vector pPICZ*α*A (Invitrogen, Carlsbad, CA, USA). Recombinant Bm91 plasmids were cloned into *E. coli *DH5-*α*  competent cells. The antigen was adjuvated with Montanide ISA 50 V containing anhydromannitoletheroctodecenoate in mineral oil (Seppic, Paris, France). For the immunizations, doses of 2 mL containing 200 *μ*g of the recombinant protein were prepared one day prior to use with an Ultra-Turrax T8 high-speed homogenizer (IKA Labortechnik, Staufen, Germany). The control and adjuvant group received 2 mL doses of phosphate buffered saline (PBS) and Montanide ISA 50 V, respectively. The animals were intramuscularly inoculated with a primary dose (week 0) followed by two doses at 3-week intervals (week 3 and 6) and a fourth immunization six months (week 26) after primary immunization (ppi). 

### 2.3. Blood Collection

Blood was collected weekly from the jugular vein into two sterile tubes for a period of three months ppi and for one month after the fourth immunization (week 26–30). The tube for serum collection contained clot activator and the serum was separated one day after collection and stored at −20°C until further analysis. Furthermore, the health status of the animals was monitored by determining the packed cell volume (PCV, %) and the hemoglobin content (Hb, g/dL) by routine laboratory methods. Therefore, blood was collected into K_3_-EDTA tubes (except week 11).

### 2.4. Anti-Bm91 Antibody ELISA

The anti-Bm91 antibody level was determined by indirect enzyme-linked immunosorbent assay (ELISA) according to Jittapalapong et al. [25] using the Thai recombinant Bm91 protein as antigen. Briefly, microtiter plates were coated overnight at 4°C with 3 *μ*g/well of antigen diluted in 0.1 M carbonate coating buffer (pH 9.6). After washing five times with PBST (0.05% (w/v) Tween-20 in PBS), the free binding sites on the plates were blocked with 0.1% bovine serum albumin in PBS (100 *μ*L per well) for 2 h at room temperature (RT). Once the plates were washed again with PBST, 100 *μ*L of sample sera in a 1 : 100 dilution were added to each well and incubated for 2 h at RT. After washing as described above, 100 *μ*L of peroxidase-conjugated goat immunoglobulins against bovine immunoglobulins (ICN, Aurora, USA) diluted 1 : 5,000 were added to each well. The plates were incubated for another 2 h at RT before another washing step followed. The antibody-antigen complexes were visualized with 100 *μ*L per well of 0.05% 2,2′-azino-di-[3-thylbenzthiazoline sulfonate] (ICN, Aurora, CO, USA) and 0.03% hydrogen peroxide. Reactions were stopped after 7 min by adding hydrochloric acid. Optical density (OD) measurements were performed using a TECAN Sunrise ELISA reader (TECAN Trading AG, Groding, Switzerland) at a wave length of 450 nm. Mean OD values were calculated from duplicates of each sample. Positive and negative controls without antigen, primary antibody, secondary antibody, and substrate were simultaneously measured to ensure that the colorimetric reaction was due to the formation of the antigen-antibody complex and not to nonspecific reactions. 

### 2.5. Western Blot Analysis

In order to confirm that the anti-Bm91 antibodies invoked by the immunization bind to *R. (B.) microplus *proteins, a multiscreen Western blot analysis was performed for the sera obtained weekly from one immunized animal. A Mini-PROTEAN II multiscreen apparatus (Bio-Rad, Hercules, CA, USA) was used. For the analysis the Bm91 antigen preparation, which was used for the immunizations, was subjected to 10% sodium dodecylsulphate polyacrylamide gel electrophoresis (SDS-PAGE). Proteins were denatured for 4 min at 95°C in loading buffer and resolved at 200 V for 40 min under reducing conditions. Afterwards, proteins were transferred to a polyvinylidene difluoride (PVDF) membrane and incubated with the weekly taken serum samples diluted 1 : 100 at 100 V for 1.5 h. After blocking with TTBS (50 mM Tris-HCl, 150 mM NaCl, 0.05% (w/v) TWEEN-20, pH 8.0), the blot was incubated overnight at RT. Prior to the incubation with peroxidase-conjugated antibovine immunoglobulins (Amersham Biosciences, Piscataway, NJ, USA) at RT, the blot was washed three times with PBST. Finally, the blot was incubated with antibovine conjugate as secondary antibody diluted 1 : 5,000 in TTBS for 1 h at RT after it was washed as described for the preliminary step. After incubating with the secondary antibody, three washing steps with PBST followed. The positive signals were visualized by adding DAB (diaminobenzidine, Sigma, USA) substrate. Protein standards represented 15–250 kDa (Bio-Rad, Hercules, CA, USA). 

### 2.6. Tick Collection and Reproductive Tick Performance

All the cattle were kept on the same tick-infested pasture and were naturally infested with *R. (B.) microplus *larvae, nymphs, and adults. In order to evaluate the effect of the Bm91-immunization on the reproductive performance, standard female *R. (B.) microplus *ticks (4.5–8 mm) were collected daily after the third and the fourth immunizations for a total period of 10 weeks. Standard female *R. (B.) microplus* ticks are those that are destined to complete engorgement within the following 24 h [26]. After recording the engorged weight, ticks were kept individually in tick chambers in a photoperiod with 12 h of light at 30 ± 5°C and 70–80% relative humidity. Approximately three weeks after tick collection, when oviposition was completed, the egg mass oviposited by each female was weighed. The egg viability was checked visually six weeks after collection and if less than 50% of the tick larvae hatched during this period the batch was recorded as nonviable. The reproductive efficiency index (REI) was calculated by using the following formula proposed by Bennett [27]: REI = [Egg mass weight (mg)/tick weight (mg)] × 100.

### 2.7. Statistical Analysis

Data were statistically analyzed with the software SAS, version 9.3 [28], and a repeated measures ANOVA over time (MIXED procedure) was used. The analysis provides  *P*  values for differences between treatments, differences over time, and the interaction between treatment and week. Chosen by Akaike's information criterion repeated measures on a given animal were assumed to have a compound symmetry structure. This structure was compared to the unstructured covariance matrix and the first-order antedependence structure. The approximation for the denominator degrees of freedom was done after Kenward and Roger [29]. The ELISA values were adjusted to an OD value of 0 at the onset of the study and the results are presented as least squares means (LSM) ± standard error (SE). Multiple comparisons were done with the Tukey-test. Differences of  *P* < 0.05  were considered as statistically significant. Tick number, tick weight, percentage of ticks ovipositing, REI, and egg viability were analyzed with the GENMOD procedure. A Poisson distribution and a logarithmic link function were assumed. Week was included as the repeated effect and groups were compared by  *χ* ^2^-test (*P* < 0.05). The average daily gain was calculated by regression analysis using the REG procedure. Body temperature, PCV, and Hb were analyzed by repeated measures ANOVA using the MIXED procedure. Group, week, and group∗week interaction were included as fixed effects and week as the repeated effect for each animal. LSMs (± pooled standard error, PSE) were compared by the Tukey-test (*P* < 0.05). If the standard error differed between the groups, the highest standard error is given as PSE. 

## 3. Results 

### 3.1. Anti-Bm91 Antibody Level

**Figure 1:**
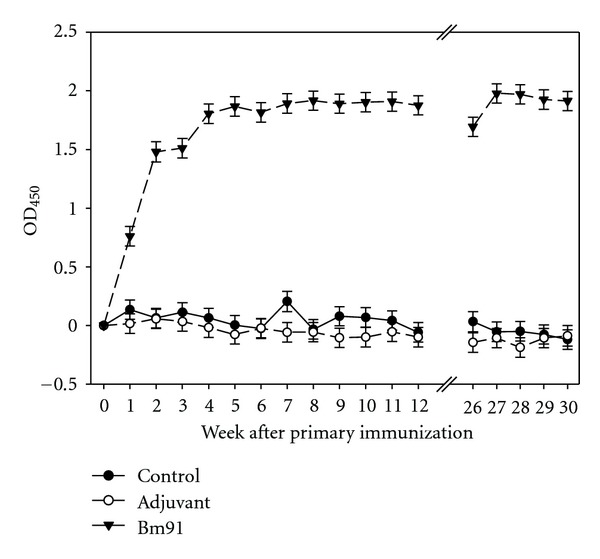
Antibody responses of *Bos indicus* cattle immunized with the antigen Bm91 and injected with saline (control) and adjuvant, respectively. Values were measured by ELISA and are expressed as optical density_450_ (OD_450_) value (LSM ± S.E.;  *N* = 6). Values were adjusted to week 0. Immunizations were given in week 0, 3, 6, and 26.

Previous to the first immunization the sera of all cattle were assayed by ELISA for anti-Bm91 antibodies and were found seronegative (Figure 1). The anti-Bm91 antibody level of the control and the adjuvant group remained seronegative throughout the entire observation period. In contrast, the immunized animals developed a strong and specific humoral immune response characterized by high anti-Bm91 IgG levels. A rapid increase of the level was observed following the primary immunization and both the second and third treatments resulted in a further rise. A stable level was reached after the third immunization dose and was maintained until week 12 ppi. This level was about 2 OD units higher in the Bm91 group when compared to the control and adjuvant group, respectively. Until week 26 the anti-Bm91 ELISA values decreased slightly. Following the fourth immunization, a moderate increase was recognized. This level remained unaltered until the end of the trial in week 30. 

### 3.2. Western Blot Analysis

**Figure 2:**
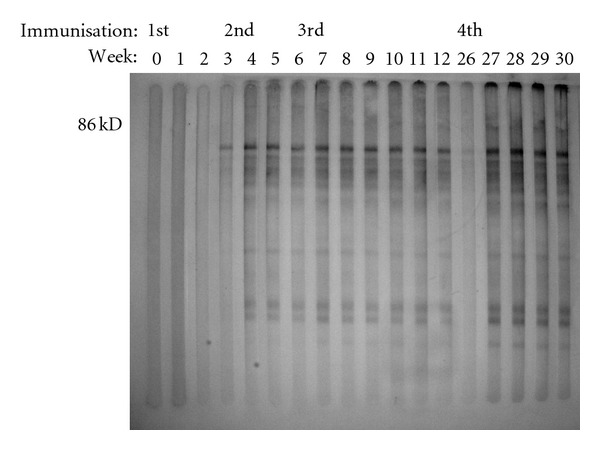
Multiscreen Western blot of sera from one Bm91-immunized animal using the Bm91 antigen formulation.

The Western blot analysis, which was exemplarily done for one Bm91-immunized animal, demonstrated that the weekly-taken immune sera of this animal bound to the proteins used in the Bm91 antigen formulation. A band at a molecular weight of 86 kDa, which is consistent with the recombinant Bm91 protein from the Thai *R. (B.) microplus *strain, was visible (Figure 2). This band was recognized from week 2 ppi on until the end of the study. In week 26 the intensity of the reaction was reduced. Furthermore, the analysis indicated that in preimmune sera from the same immunized animal this band at 86 kDa was not present.

### 3.3. Tick Parameters

The different parameters recorded from the naturally infested *R. (B.) microplus* ticks are given in Table 1. The number of ticks collected did not differ significantly between the Bm91, control, and adjuvant groups. Within the three groups a high animal-to-animal variation was recorded. No significant group difference was noticed for the tick weight. In comparison to the control group the proportion of ovipositing ticks was reduced by 5% in the adjuvant group (*P* < 0.05). An effect of the immunization was recorded on REI and egg viability. Compared to the control group, a reduction of 6% and 8%, respectively, was observed for these two parameters in the immunized group (*P* < 0.05).

### 3.4. Weight Gain, Body Temperature, and Blood Parameters

**Figure 3:**
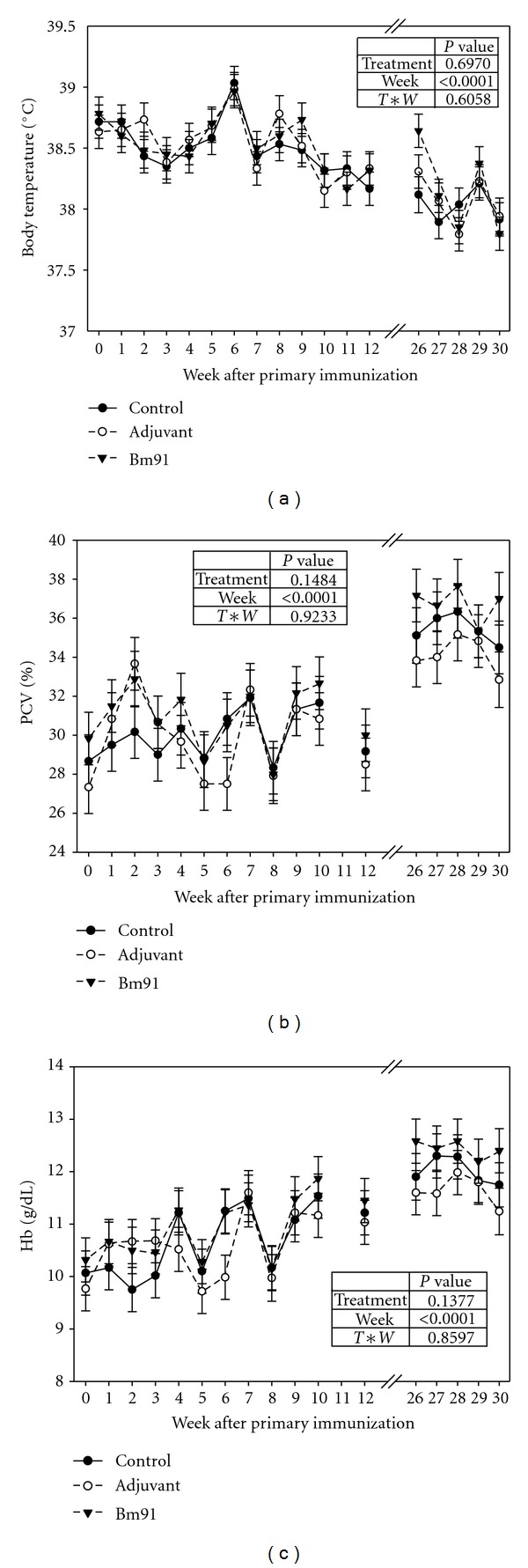
Body temperature (a), PCV (b), and Hb (c) of *Bos indicus *cattle immunized with the antigen Bm91 and injected with saline (control), and adjuvant, respectively (LSM ± S.E.;  *N* = 6).

The average daily weight gain of the cattle was 203, 174, and 175 g/d (PSE = 10.7) for the control, adjuvant, and Bm91 group, respectively. Differences between the groups were not significant (*P* > 0.05). The mean body temperature ranged between 37.8°C and 39.0°C (Figure 3(a)). Neither for treatment nor for the interaction between treatment and week a significant effect was observed by repeated measures ANOVA. The course of PCV and Hb is given in Figures 3(b) and 3(c). Both parameters varied within the reference intervals proposed by Kramer [30]. After the fourth treatment (week 26) higher values were observed than during the weeks 0 to 12 ppi. For neither of the parameters the group effect as well as the interaction between group and week were significant (*P* > 0.05). In contrast, week as the repeated effect was significant for all parameters indicating that the values changed over time.

## 4. Discussion

Given the benefits of a recombinant protein vaccine over traditional chemical tick control, it is important to explore the potential of the immunization with the antigen Bm91 under field conditions of natural tick infestation. *R. (B.) microplus* is one of the most important arthropods impairing livestock—especially cattle—production in tropical and subtropical countries. The commercial anti-tick vaccines, Gavac and TickGARD, both contain the recombinant antigen Bm86, whereas each is from a different strain of *R. (B.) microplus* [31–33]. Studies investigating Bm86 under controlled conditions with artificial *R. (B.) microplus *infestations demonstrated that the immunization reduced the number and the weight of engorging female ticks, their egg laying capacity, and their fecundity [13, 14]. Following the isolation of Bm86 a number of other tick-protective antigens were isolated. Bm91 is one of these tick-protective antigens, which was tested in an immunization trial in combination with Bm86 [34]. Even though Bm91 was not included into the commercial anti-tick vaccines, it is a candidate for controlling cattle ticks effectively. Nevertheless, whenever tick-protective antigens are evaluated the sequence variation of *R. (B.) microplus *strains has to be taken into consideration. In dependence of the tick strain the antigen is derived of, the effect on the tick reproduction may differ substantially [22]. 

Thus, our overall aims of the study were to evaluate the humoral immune response against the Bm91 antigen in *B. indicus *cattle and to assess the efficacy on the reproductive performance of *R. (B.) microplus *under field conditions of natural infestation. Through deriving the antigen from indigenous ticks, a divergence between the recombinant protein of the vaccine and the native antigen of the tick could be excluded in the present study. 

The antibody titer was demonstrated to be the major determinant of the efficacy of anti-tick vaccines [14]. A broad correlation was found between the titer of antibodies to Bm86 and the vaccine efficacy [35]. This positive correlation of the antibody titer with the vaccine efficacy could be confirmed not only for Bm86 [20, 21], but also for Bm91 [34], and Bm95 [36]. In conclusion, it permits the evaluation of the vaccine efficacy through the measurement of antibody titers in immunized animals [37]. Given the primary immunization followed by two booster doses with three weeks in-between, the time scheme of the present trial is consistent with other vaccination trials. The immunized animals developed a strong and specific humoral immune response expressed by high anti-Bm91 antibody levels, which increased immediately after the first immunization. Contrary to the results of the present study, the most pronounced rise in anti-Bm86 and anti-Bm95 titers was not recorded after the first but after the second immunization in the study of García-García et al. [38]. The steady decrease of the Bm86 and Bm95 titres following their peaks 2 weeks after the second immunization is not in agreement with the course of Bm91 in the present study. A similar course with the peak being followed by a strong decrease was shown in the study of Patarroyo et al. [39] using synthetic peptides derived from Bm86. Low responses to the primary immunization with TickGARD^Plus^ and Gavac were also measured by Andreotti [20]. In agreement with the aforementioned studies, the antibody level peaked two weeks after the second immunization. A distinct decrease followed the peak. A low response to the first two immunizations (week 0 and 4) was in the retrospective analysis of the use of Gavac in the field [40]. As observed under controlled conditions by Rodríguez et al. [13], a strong increase of the antibody level was apparent after the third treatment (week 7). A strong decline followed the peak two weeks after this dose. Anti-Bm95 antibody levels showed a similar course with a strong rise following the third immunization in the study of Kumar et al. [36]. The short duration of protective titers in some animals in the study of Jonsson et al. [18] is not consistent with the results found here. Contrarily, the anti-Bm91 antibodies were maintained at a high level throughout the observation period. Particularly, the high level measured prior to the fourth immunization six months ppi contrasts to other studies. Within six months antibody levels decreased to preimmunization levels in the study of García-García et al. [38]. A similar decline was described by Rodríguez et al. [13], Valle et al. [40], and Andreotti [20]. Comparing three immunization schemes with Gavac^Plus^, Vargas et al. [41] found that the number of vaccinations and the interval between the treatments did not affect the antibody level. The possibility of reducing the number of immunizations was therefore suggested. In general, higher variations within immunized groups than found here are reported in the literature [18, 20]. In broad agreement with the course of the anti-Bm91 level of the present study are the ones for Bm86 and Bm95 obtained by Jittapalapong et al. [42].

The ELISA results could be confirmed by the Western blot analysis. This analysis indicates that Bm91 is immunogenic and induced antibodies that bind to *R. (B.) microplus *midgut proteins. The 86 kDa band, which is consistent with recombinant Bm91 from the Thai *R. (B.) microplus *strain, was recognized from the third week ppi onwards. Hence, the glycoprotein elicited specific antibodies that bind to *R. (B.) microplus *proteins. Sera of cattle, which were immunized with synthetic Bm86 peptides [39] or with glycoproteins isolated from *R. (B.) microplus *and *Hyalomma anatolicum anatolicum *[43] revealed similar Western blot results than presented here.

As the ELISA and Western blot results confirmed, ticks that attached to a Bm91-immunized animal were subjected to the ingestion of anti-Bm91 antibodies with the blood meal. The ingestion of blood containing anti-tick antibodies causes the lysis of the tick digest cells resulting in the aforementioned effect on the reproductive tick performance [44]. Under artificial infestation Bm86 reduced the number of ticks and the reproductive performance by 56% and 72%, respectively [18]. Andreotti [20] found a protection efficacy of 49% and 46% for Gavac and TickGARD^Plus^, respectively. García-García et al. [38] vaccinated cattle with Bm86 and Bm95 derived from Argentinean tick strains. Thereafter, the animals were challenged with *R. (B.) microplus *larvae of either an Argentinean or an Australian tick strain. Against the Argentinean strain efficacies of 84% and 89% were calculated for Bm86 and Bm95, respectively. Contrarily, the protection against the Australian ticks was 0% and 54% for Bm86 and Bm95, respectively. In a recent study, Almazán et al. [21] found Bm86 to reduce the number of ticks, the egg weight, the oviposition, and the egg fertility by 51%, 5%, 14%, and 6%, respectively. Similar vaccine efficacies were also reported for the Bm86 homologues Ba86, Bd86, and Haa86 [19, 21, 45–47]. In general, the most pronounced effect of anti-tick vaccines is rather seen on the reproductive tick performance than on the direct reduction of engorging ticks. This emphasizes the prophylactic use of vaccines with the greatest effect seen in a gradual reduction of the tick population. Concordant values for Bm91 are not found in the literature, because this antigen was not tested as a stand-alone antigen in immunization trials. In comparison to other immunization trials, the effect of Bm91 on the tick parameters, particularly on REI and egg viability, found in the present study was low. Regarding the number of ticks, it has to be taken into account that the animals were naturally infested with *R. (B.) microplus. *The high variation of tick counts between animals indicates the skewed distribution of ticks in a herd as shown previously [18]. The study of Jittapalapong et al. [48] was conducted under comparable conditions. Nevertheless, the tick infestation was considerably higher. As for the tick number, an effect of Bm91 on the tick weight and the percentage of ticks ovipositing were not recorded. However, the 6% and 8% reduction of the REI and the egg viability, respectively, in comparison to the control group indicates that Bm91 affected the reproductive tick performance. Another issue, which has to be addressed in the present study, is that it was conducted under field conditions of natural *R. (B.) microplus *infestation. Generally, the natural tick infestation in field experiments varies depending on a variety of factors, mainly climatic, and is difficult to predict [14, 49]. The primary immunization was immediately given before the onset of the rainy season; however, the tick infestation during the following rainy season was lower compared to other studies with natural infestation [48]. 

The weight gains observed here reflect the influence of the season. With the duration of the study, the feed quality and quantity improved. Due to the low tick infestation, an effect of the immunization on the weight gain could not be expected. The decreasing body temperature in the course of the study reveals the exposure of the animals to the environmental conditions. This could also be noticed for PCV and Hb indicating the increasing health status due to the seasonal conditions. The immunization did not influence the health status of the animals. Therefore, it can be recommended that the immunization can be given independent of the season, though the high adaptation of the *B. indicus *animals used in the present study to the local environmental conditions has to be mentioned here. In order to gain the greatest effect, the immunization is recommended before the onset of the rainy season to ensure that the antibody level has reached a stable level during the rainy season when the highest tick infestation is usually observed. 

In conclusion, a strong and long-lasting immune response could be invoked by recombinant Bm91, which was derived from a local *R. (B.) microplus *strain. All immunized cattle responded to such an extent that the studied immunization scheme with three primary immunizations and the booster dose after six months can be recommended as appropriate in order to maintain adequate antibody levels. Although a positive effect on the REI and the egg viability was observed under field conditions, vaccine efficacies observed in other studies after artificial tick infestation were not reached. Despite the limited effect of Bm91 on the tick reproduction, the high immunogenicity of the antigen under field conditions shown here warrants its further evaluation. Though the use of Bm91 as part of an integrated tick control strategy requires more pronounced effects on the tick reproduction. Therefore, the evaluation under field conditions of natural *R. (B.) microplus* infestation in combination with other antigens is recommended.

## Figures and Tables

**Table 1:** Number, weight, oviposition, and hatching of naturally infested *R. (B.) microplus* ticks collected from *Bos indicus* cattle immunized with the antigen Bm91 and injected with saline (control) and adjuvant, respectively (*N* = 6).

Parameter	Control	Adjuvant (% reduction)*	Bm91 (% reduction)
Tick number (mean ± SD)**	90 ± 64^NS^	62 ± 40	81 ± 37
Tick weight in mg (mean ± SD)	84.0 ± 32.3^NS^	80.4 ± 30.5 (4)	84.0 ± 30.2 (0)
Ticks ovipositing (%)	95.3^a^	91.7^b^ (5)	94.3^ab^ (1)
REI (%)***	38.8 ± 9.7^a^	38.5 ± 9.6^a^ (1)	36.6 ± 9.2^b^ (6)
Egg viability (%)	84.4^a^	84.7^a^ (−1)	77.8^b^ (8)

*The percent reduction was calculated with respect to the control group.

**Values within the same row differ significantly (*P* < 0.05; *χ* ^2^-test).

***Reproductive efficiency index.

^NS^ *P* > 0.05.

^a, b^Values within a line with different letters differ significantly (*P* < 0.05).
